# A Compact SLED Light Source Driver Module for Optical Coherence Tomography Applications

**DOI:** 10.3390/s26072084

**Published:** 2026-03-27

**Authors:** Yuanhao Cao, Feng Liu, Jianguo Mei, Qun Liu, Biao Chen

**Affiliations:** 1 College of Optical Science and Engineering, Ningbo Global Innovation Center, Zhejiang University, Ningbo 315100, China; 22460537@zju.edu.cn; 2 Ningbo Ming Sing Optical R&D Co., Ltd., Ningbo 315000, China; liuf@nbmingsing.com (F.L.); mjggood@163.com (J.M.)

**Keywords:** superluminescent light-emitting diode (SLED), lightweight design, constant-current drive, temperature control, analog PID

## Abstract

Optical coherence tomography (OCT) is a non-invasive, high-resolution imaging technique widely used in medical diagnosis, biomedical research and other fields. It plays an important role in the early detection and accurate diagnosis of diseases. The superluminescent light-emitting diode (SLED) is the ideal light source for OCT systems, where the stability of its drive current and operating temperature directly determines the imaging quality of OCT. Existing driving and temperature control schemes for similar light sources predominantly rely on microcontrollers or field programmable gate arrays (FPGAs), a reliance which often results in complex system architectures and difficulties in balancing simplicity with control precision. To address these issues, a stable and compact SLED source driver module designed for OCT was developed in this study, integrating both a constant-current drive circuit and a temperature control circuit. The negative feedback control and improved current-limiting protection are employed in the constant-current drive circuit to maintain stable SLED operation and reduce the circuit footprint. A miniature dedicated temperature control chip is adopted in the temperature control circuit. The operating temperature of the SLED is acquired by linearizing the negative temperature coefficient (NTC) thermistor value and regulated through a proportional-integral-derivative (PID) compensation circuit. The size of the fabricated module (including casing) is less than 10 × 8 × 3 cm^3^. Experimental results show that the driver module achieves a drive current control accuracy of 0.1% and a temperature control accuracy of 0.01 °C. The output optical power fluctuation is less than 0.005 mW and the average axial resolution for OCT is 6.5992 μm with a standard deviation of 0.0107 μm. This light source driver module successfully balances control precision with structural simplicity, demonstrating excellent applicability in OCT systems.

## 1. Introduction

The superluminescent light-emitting diode (SLED) exploits amplified spontaneous emission while suppressing optical feedback. This mechanism confers broader spectral bandwidth than laser diodes (LDs) and higher output power than light-emitting diodes (LEDs) [1,2,3]. These intermediate optical characteristics make SLEDs indispensable for fiber-optic gyroscopes (FOGs) [4,5] and optical coherence tomography (OCT) systems. OCT is a non-invasive, high-resolution imaging technique for biological tissues and materials, which has gained widespread biomedical adoption, particularly in ophthalmic diagnostics [6]. Its performance is characterized by multiple metrics including resolution, sensitivity, and penetration depth, with axial resolution being the predominant determinant of imaging quality. SLEDs serve as an ideal light source for OCT systems due to their high power, broad spectrum, and low coherence, which collectively enhance axial resolution. However, the performance of such optoelectronic devices is highly sensitive to operating conditions. Minor fluctuations in drive current or operating temperature can degrade output stability. OCT systems impose stringent requirements on source stability: optical power directly affects imaging depth and brightness, while central wavelength and spectral bandwidth critically determine axial resolution [7]. Consequently, precise control of both drive current and operating temperature is essential for SLEDs employed in OCT applications. Considering the limited internal space of OCT devices, the corresponding light source modules used should be as compact as possible while avoiding excessive complexity.

To date, researchers have investigated driving and temperature control strategies for similar light sources. Zhang et al. [8] implemented a microcontroller-based system employing variable-domain fuzzy proportional-integral-derivative (PID) control, achieving ±0.1 °C precision across a broad temperature range (−12 °C to 120 °C) through pulse-width modulation. Liu et al. [9] developed an FPGA-based platform that reduced wavelength drift by 65% while maintaining temperature errors below 0.4 °C via real-time state machine switching. Wang et al. [10] combined the ADN8834 temperature controller with the LTC2377 analog-to-digital conversion chip and a field programmable gate array (FPGA) to realize incremental digital PID control, achieving a control accuracy of 0.005 °C. Zhai et al. [11] demonstrated genetic algorithm-optimized PID parameters for laser diode temperature control through simulation. Xu et al. [12] achieved 0.01 mA current regulation and maintained temperature fluctuations in the range of ±0.1 °C using deep negative feedback circuits coupled with fuzzy adaptive PID and H-bridge drivers. Miao et al. [13] attained 0.005 °C precision via STM32-based fuzzy PID control alongside power amplifier-driven current sources. Chen et al. [14] utilized the MAX1978 to reach a temperature control precision of 0.01 °C while extending the linear operating range of current sources to 1 A. Zhao et al. [15] developed a current driver featuring closed-loop negative feedback with linear soft-start and current-limiting circuits, coupled with a fuzzy PID-controlled thermal system. The entire system achieved a current control precision of 0.01 mA and a temperature control precision of 0.005 °C.

Existing studies illustrated above demonstrate remarkable precision in controlling driving current and temperature, yet most solutions inevitably rely on microcontrollers or FPGAs with overly complex system architectures [16,17], making it difficult to strike a balance between simplicity and control accuracy. For example, the method proposed by Zhao et al. [15] achieves good control accuracy in both current and temperature, but the overall structure is not well integrated. The entire module consists of three parts: a current driver board, a temperature control board, and a microcontroller, each of which has a relatively large volume. The method proposed by Yang et al. [18] uses an FPGA as the control core and also includes three parts: a current driver board, a temperature control board, and an FPGA. The wiring in the entire module is messy. Furthermore, most driving and control studies targeted semiconductor lasers, with comparatively fewer investigations specifically targeting SLEDs.

In this paper, the design and working principle of a stable, compact SLED light source driver module for OCT applications are presented. The system integrated a compact constant-current drive circuit and temperature control circuit based on a monolithic temperature control chip. The constant-current drive circuit was mainly composed of a negative feedback circuit and a protection circuit, while the temperature control circuit, based on closed-loop negative feedback control, was composed of a temperature detection circuit and a temperature compensation circuit. This study established a mathematical model based on mechanistic analysis of the temperature control subject, then elaborated on the working principle of the analog PID compensation circuit and verified its reliability through simulation. The improved current-limiting protection principle and the temperature control implemented by fully analog circuit further reduced the overall system size. The light source driver module designed in this study achieves a balance between control accuracy and structural simplicity, thus exhibiting superior applicability in OCT systems.

## 2. The Effect of Driving Current and Temperature on the Output Light of SLED

As a semiconductor optoelectronic device, SLED features a current-driven P-N junction as its core component. The output optical power, wavelength, and bandwidth of the SLED are directly dependent on the driving current. Additionally, temperature exerts a significant influence on the physical properties of the P-N junction. When the driving current remains constant, variations in temperature will induce corresponding changes in the output optical power and wavelength of the SLED. To clarify the performance of the SLED, its current and temperature characteristics were tested, and the resulting characteristic curves are presented below.

**Figure 1:**
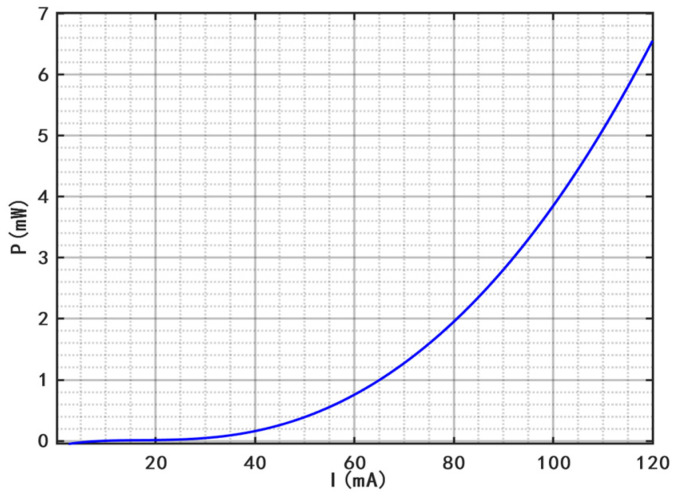
Power-current characteristics of SLED.

**Figure 2:**
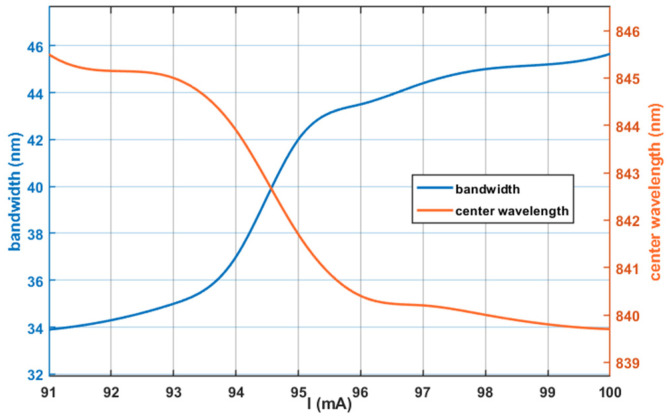
Bandwidth-current and center wavelength-current characteristics of SLED.

**Figure 3:**
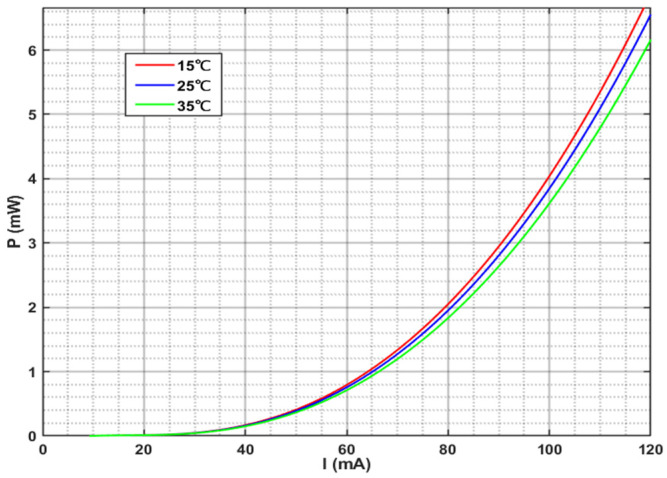
Power-current characteristics of SLED at different temperatures.

**Figure 4:**
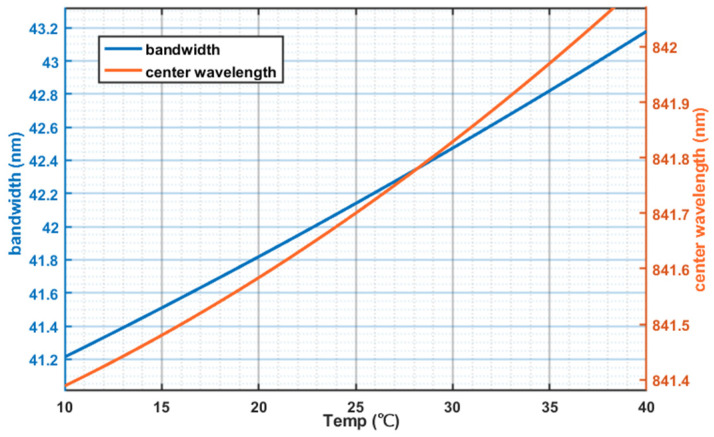
Temperature effect on the bandwidth and center wavelength.

The optical power-current characteristic is illustrated in Figure 1. At a constant temperature of 25 °C, the output optical power increases with the driving current. Furthermore, within the threshold current range, the growth rate of the optical power increases with the increase in the driving current. Figure 2 shows the curves of the output optical bandwidth and center wavelength as a function of the driving current. With the increase in the driving current, the output optical bandwidth broadens, and the central wavelength shifts gradually toward the short-wavelength direction. Figure 3 presents the optical power versus driving current curves at different temperatures (15 °C, 25 °C, 35 °C). At the same driving current, the output optical power decreases with the elevation of temperature. Notably, the reduction in output optical power leads to an increase in heat dissipation, which is prone to further raise the operating temperature, thereby forming a vicious cycle. Figure 4 reflects the relationship between the output optical bandwidth, center wavelength and temperature. With the driving current kept constant, the output optical bandwidth increases moderately, and the center wavelength shifts toward the long-wavelength direction as the temperature rises. Consequently, maintaining the stability of the driving current and operating temperature is critical for enhancing the stability of the SLED output light.

## 3. Design of Constant-Current Drive and Temperature Control

Our design incorporates both constant-current drive and temperature control subsystems on a single circuit board. The constant-current module employs a feedback control circuit as its core component, augmented with protection mechanisms to prevent abnormal light source degradation. For temperature regulation, the system features detection and compensation control circuits utilizing the ADN8834 chip, which integrates multiple amplifiers and metal-oxide-semiconductor field-effect transistors (MOSFETs) to significantly reduce system footprint while enhancing operational practicality.

### 3.1. Constant-Current Drive Circuit Design

**Figure 5:**
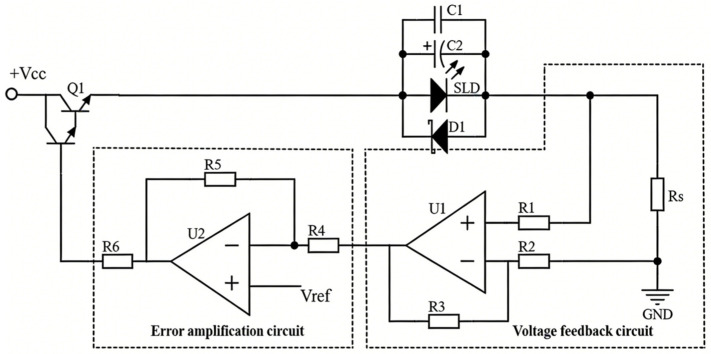
Constant-current drive circuit.

Unlike the existing method of adjusting the conduction level of a MOSFET to control the drive current magnitude [15], the use of a Darlington transistor to control current allows for lower control voltages while avoiding the risk of reverse conduction in MOSFETs. Additionally, based on the negative feedback principle, the precise adjustment of the drive current is achieved. The basic circuit implementation is shown in Figure 5.

This circuit can be divided into two functional blocks: a voltage feedback circuit and an error amplification circuit. The current flowing through the SLED is converted into a voltage output via current-to-voltage (I-V) conversion using resistor *R_s_*. Operational amplifier U1 constitutes a voltage amplifier that amplifies the I-V-converted input voltage with a gain of (1 + *R*_3_*/R*_2_). The amplified voltage is then fed into the inverting input of U2, which functions as an error amplifier, while its non-inverting input connects to a reference voltage. When the SLED drive current fluctuates, U2’s output dynamically adjusts to suppress this variation. For example, when the drive current decreases, the converted and amplified feedback voltage likewise decreases. Since the reference voltage remains constant, the output of the error amplifier increases accordingly. This leads to a rise in the base current of the Darlington transistor, thereby increasing the SLED drive current. This mechanism promptly compensates for the initial current reduction, ultimately maintaining stable drive current and achieving the goal of constant-current driving. The magnitude of the drive current can be easily tuned by changing the reference voltage *V_ref_*.

To avoid excessive output light intensity caused by an overly large driving current during operation, which could damage the human eye, and considering that SLED chips are extremely fragile and prone to damage from excessive driving currents, the driving circuit should be equipped with current-limiting protection. Existing current-limiting protection circuits essentially introduce another constant-current drive circuit [15,18], which sets a limiting voltage *V_lim_*. By comparing *V_lim_* with the reference voltage *V_ref_*, the MOSFET’s conduction level is reduced to obtain a safer drive current. However, this protection method increases the overall circuit scale. For our design emphasizing compactness, miniaturization and reliability, resistor *R_s_* is employed for not only detecting current magnitude but also limiting excessive current. The maximum drive current *I_max_* can be calculated as:

*I_max_* = *V_cc_* − *V_ce_* − *V_sled_*/*R_s_* (1)

So, the value of *R_s_* is

*R_s_* = (*V_cc_* − *V_ce_* − *V_sled_*)/*I_lim_* (2)

where *V_ce_* is the voltage between the collector and emitter of the Darlington transistor, which can be estimated using its saturation state value; *V_sled_* is the voltage across the SLED, increasing with drive current; and *I_lim_* is the preset current limit. Once *I_lim_* is set, *V_sled_* is determined, allowing the estimation of *R_s_*. In addition, a capacitor C1 with small capacitance, an electrolytic capacitor C2 with large capacitance, and a transient diode D1 are connected in parallel across the SLED. Capacitor C1 is used to filter out high-frequency noise in the circuit, capacitor C2 serves to suppress sudden voltage changes across the SLED, and diode D1 protects the SLED from electrostatic damage.

### 3.2. Constant Temperature Control Circuit Design

**Figure 6:**
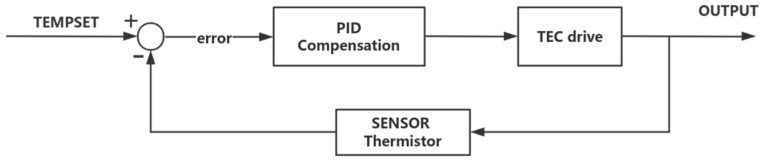
Block diagram of temperature control process.

Temperature stabilization is achieved through a closed-loop negative feedback system. The control process comprises three stages: detection, control, and execution [19]. For most SLED devices, negative temperature coefficient (NTC) thermistor and thermoelectric cooler (TEC) are pre-integrated within the same package as the temperature sensor and actuator, respectively, facilitating precise regulation of the operating temperature. During the temperature detection stage, the NTC thermistor monitors the real-time temperature of the device. This measured value is compared with the setpoint temperature, and the resulting error signal is amplified and fed into the compensation circuit. The compensation circuit then processes this signal through PID control to generate a corrective output, which drives the TEC for cooling or heating, thereby achieving temperature control. The system control flow is illustrated in Figure 6.

#### 3.2.1. Temperature Detection Circuit

**Figure 7:**
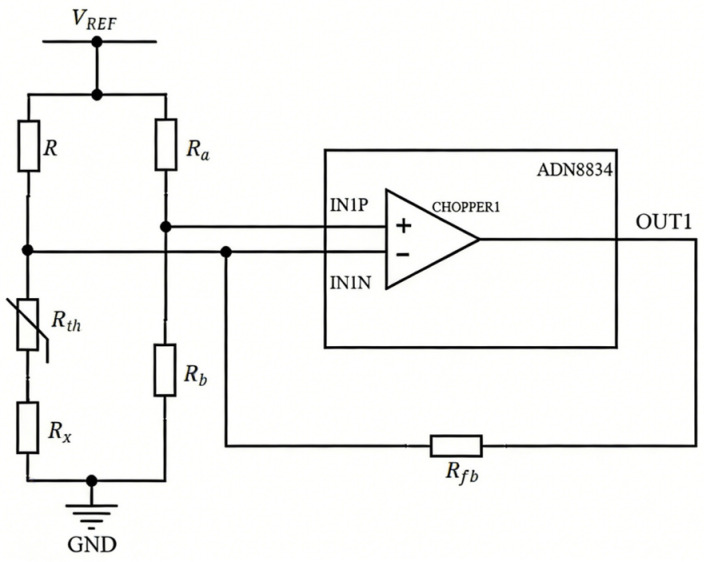
Temperature detection circuit.

The resistance-temperature characteristic of the NTC thermistor follows a logarithmic relationship. This inherent nonlinearity, where sensitivity varies with temperature, prevents direct generation of the linear voltage output required for subsequent control. We utilize CHOPPER1, a spare operational amplifier provided by the ADN8834 chip, to implement the circuit shown in Figure 7. By connecting a compensation resistor of appropriate value in series with the NTC thermistor, an improved linearity output is achieved. The resistance value of the compensation resistor *R_x_* and *R* is calculated using the following formula:

(3) $$R_{x}=\left(\frac{R_{LOW}R_{MID}+R_{MID}R_{HIGH}-2R_{LOW}R_{HIGH}}{R_{LOW}+R_{HIGH}-2R_{MID}}\right)$$

(4) $$R=R_{x}+R_{th\_25}$$

where *R_th__*_25_ denotes the resistance of the NTC thermistor at 25 °C, which is set as the optimal operating temperature. *R_LOW_*, *R_MID_*, and *R_HIGH_* represent the resistance values at temperatures *T_LOW_*, *T_MID_*, and *T_HIGH_*, respectively; *T_LOW_* and *T_HIGH_* are the endpoints of the temperature range, while *T_MID_* is the mean temperature. In cases where only the β-constant of the NTC thermistor is available, the resistance at temperature *T* is calculated as:

(5) $$R_{th}\;=\;R_{th0}\cdot exp\left\{\beta \left(\frac{1}{T}\;-\;\frac{1}{T_{0}}\right)\right\}$$

where *R_th_* represents the resistance of the NTC thermistor at temperature *T*, and *R_th_*_0_ denotes its resistance at reference temperature *T*_0_.

In the circuit, resistors *R_a_* and *R_b_* form a voltage divider to provide a reference voltage to the non-inverting input of the amplifier. Simultaneously, resistor *R* combines with *R_x_* and *R_th_* to create another voltage divider, generating a temperature-dependent voltage signal that feeds into the amplifier’s inverting input. The feedback resistor *R_fb_*, together with these voltage divider networks, constitutes the temperature detection amplification circuit. Consequently, the output voltage can be expressed as:

(6) $$V_{OUT1}\;=\;\left(\frac{R_{fb}}{R_{x}+R_{th}}\;-\;\frac{R_{fb}}{R}\;+\;1\right)\;\times \;\frac{V_{REF}}{2}$$

Through this circuit configuration, an approximately linear voltage-temperature relationship can be achieved within a specified temperature range.

**Figure 8:**
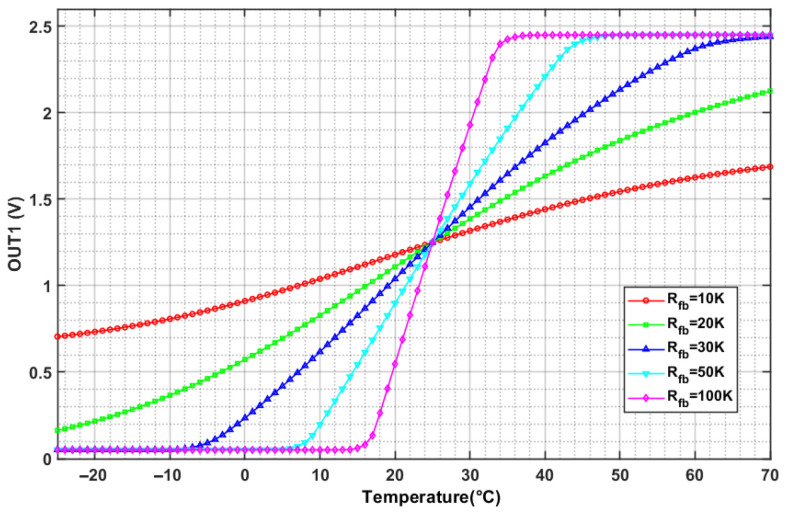
Simulation of temperature measurement circuit.

To validate the circuit’s output reliability, LTspice simulations were conducted with the NTC thermistor’s resistance-temperature characteristic defined via Equation (5). The β-constant of the NTC thermistor is 3950 and the resistance at 25 °C is 10 kΩ. Meanwhile, the parameters *R_a_*, *R_b_*, *R* and *R_x_* were set to 10 kΩ, 10 kΩ, 20 kΩ and 10 kΩ, respectively. As shown in Figure 8, when the feedback resistance *R_fb_* is set to 20 kΩ, the amplifier output demonstrates excellent linearity across the −10 °C to 60 °C temperature range.

#### 3.2.2. Temperature Control Model

It is helpful to get the thermal control system model for precise temperature regulation. The TEC itself exhibits thermal inertia. The temperature regulation process of such components can generally be characterized as a first-order inertial element [20]. Neglecting spatial temperature gradients and considering only bulk temperature variations, the heat transfer relationship derived from the thermal equilibrium equation is expressed as [21]:

(7) $$K\frac{\partial \left(T-T_{0}\right)}{\partial t}\;+\;PA\left(T\;-{\;T}_{0}\right)\;=\;∆Q$$

where *K* is the coefficient of refrigeration of the TEC, *P* denotes the heat transfer coefficient, *A* represents the heat transfer area, *T*_0_ and *T* correspond to initial and final temperatures, and *Q* signifies the heating power of the TEC.

When operating near a stable working point, the small-signal assumption allows linearization of the correspondence between variations in heat generation power ΔQ and voltage variations ΔU (U is the voltage across the TEC):

(8) ∆Q = f∆U

Substituting into the original equation and applying the Laplace transform yields:

(9) $$G\left(s\right)=\;\frac{∆T}{∆U}\;=\;\frac{f}{Ks+PA}\;=\;\frac{f}{PA}\;\cdot \;\frac{1}{\frac{K}{PA}s+1}$$

where *s* is the complex frequency. Both temperature sensing and compensation processes inevitably exhibit transmission delays. Therefore, a time-delay element is incorporated into the first-order inertial model to better characterize the system. The transfer function is expressed as:

(10) $$G\left(s\right)\;=\;K_{1}\frac{1}{T_{1}s\;+\;1}\;\cdot {\;e}^{-\tau s}$$

where *K*_1_ is the amplification factor, *T*_1_ denotes the time constant of the inertial element, and *τ* represents the lag time constant.

#### 3.2.3. Temperature Compensation Control

**Figure 9:**
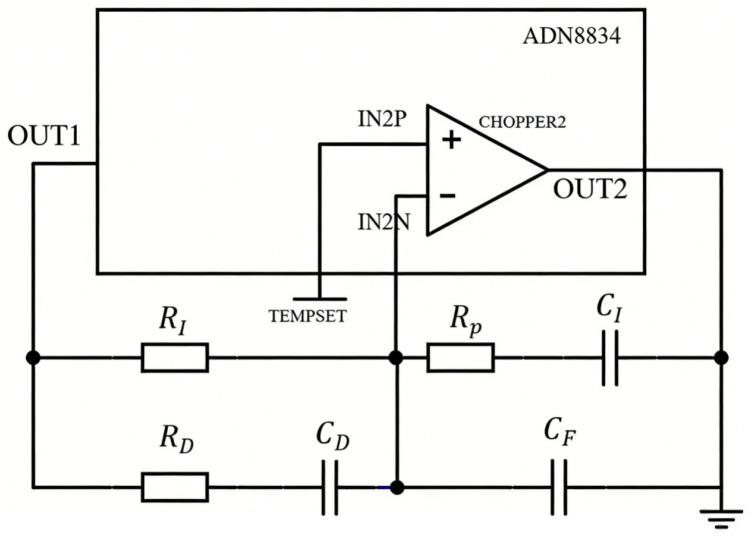
PID compensation circuit.

PID compensation controller provides effective compensation for such a thermal control model as illustrated in (10) [22,23]. The proportional amplification stage in the PID controller directly regulates system error, while the integral stage eliminates steady-state error, and the derivative stage compensates for phase lag in the thermal loop to improve response speed. Given the system’s simplicity requirements and the chip datasheet recommendation, a PID compensation circuit was designed using the chip’s internal operational amplifier CHOPPER2 as shown in Figure 9.

As shown, resistor *R_P_* functions as the feedback resistor and *R_I_* as the input resistor, collectively forming the proportional amplification circuit. The proportional gain coefficient correlates with the ratio of *R_P_* to *R_I_*. When an error exists, the output signal rapidly tracks error variations through this proportional stage. Simultaneously, capacitor *C_I_* at the output terminal combines with resistor *R_I_* to establish an integrator circuit. The error voltage gradually accumulates across *C_I_* through charging, and this cumulative effect drives continuous adjustment of the control variable until the temperature deviation reaches zero. Capacitor *C_D_* responds sensitively to voltage change rates. During rapid temperature deviations (e.g., sudden SLED chip heating), the voltage across *C_D_* reflects instantaneous change rates. The differentiator circuit formed by *C_D_* and output resistor *R_P_* proactively suppresses deviation amplification based on the error change rate. Resistor *R_D_* in series with *C_D_* increases the total impedance of the differentiation branch, limiting high-frequency current through *C_D_*. This prevents excessive sensitivity of the differentiator to high-frequency noise, thereby inhibiting noise amplification. *R_D_* also adjusts the time constant of the derivative stage to ensure appropriate differentiation intensity. The presence of *C_F_* further filters high-frequency noise, enhancing overall temperature control stability.

In the above circuit, the output at the OUT2 terminal is calculated as:

(11) $$V_{OUT2}\;=\;V_{TEMPSET}\;-\;\frac{Z_{2}}{Z_{1}}(V_{OUT1}\;-\;V_{TEMPSET})$$

In this equation, *V_TEMPSET_* represents the ideal control temperature and is input through the chip’s IN2P pin. *V_OUT_*_1_ denotes the voltage output from the thermistor after temperature sensing. Theoretically, *V_OUT_*_1_ and *V_TEMPSET_* should be equal at the target temperature. *Z*_1_, *Z*_2_ signifies the impedance network between the OUT1, IN2N, and OUT2 pins, defined by:

(12) $$Z_{1}\;=\;R_{I}//\left(R_{D}\;+\;\frac{1}{C_{D}s}\right)\;=\;\frac{R_{I}(R_{D}C_{D}s\;+\;1)}{\left(R_{I}\;+\;R_{D}\right)C_{D}s\;+\;1}$$

(13) $$Z_{2}=\left(R_{p}+\frac{1}{C_{I}s}\right)//\frac{1}{C_{F}s}=\frac{R_{p}C_{I}s+1}{(R_{p}C_{I}C_{F}s+C_{I}+C_{F})s}$$

The transfer function of the compensation circuit is thus derived as:

(14) $$G_{C}\left(s\right)\;=\;\frac{V_{OUT2}\;-{\;V}_{TEMPSET}}{V_{TEMPSET}\;-\;V_{OUT1}}\;=\;\frac{Z_{2}}{Z_{1}}$$

Substituting *Z*_1_, *Z*_2_:

(15) $$G_{C}\left(s\right)\;=\;\frac{\left(R_{p}C_{I}s\;+\;1)[\left(R_{I}\;+\;R_{D}\right)C_{D}s+\;1\right]}{R_{I}(R_{D}C_{D}s\;+\;1)(R_{p}C_{I}C_{F}s\;+{\;C}_{I}\;+\;C_{F})s}$$

Capacitor *C_F_* functions as a high-frequency filtering capacitor primarily for noise suppression. Given *C_F_* ≪ *C_I_*, the term *C_I_*/(*C_I_* + *C_F_*) approximates to 1. The transfer function simplifies to:

(16) $$G_{C}\left(s\right)\;=\;\frac{\left(R_{p}C_{I}s\;+\;1)[\left(R_{I}\;+\;R_{D}\right)C_{D}s+\;1\right]}{R_{I}C_{I}s(R_{D}C_{D}s\;+\;1)\left(R_{p}C_{F}s\;+\;1\right)}$$

**Figure 10:**
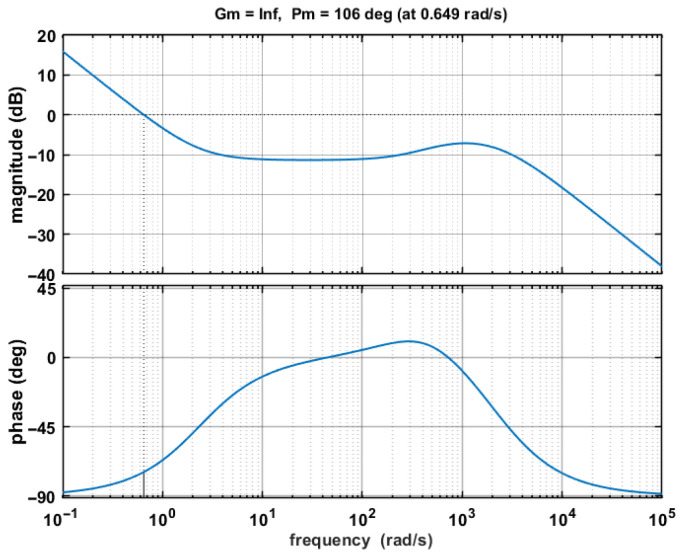
Bode plot of compensation circuit.

The reliability of the compensation circuit is ensured by observing its Bode plot via simulation, which must exhibit sufficient stability margins. To guarantee stability, the unit-gain crossover frequency must be lower than the angular frequency corresponding to the thermal time constant of the TEC and thermistor. Since this time constant is difficult to determine precisely, the initial design should maintain this frequency as low as possible. Through theoretical analysis and experimental tuning, a set of parameters was determined as listed in Table 1. The Bode plot corresponding to these parameters is shown in Figure 10. This compensation circuit demonstrates a phase margin of 106° and an infinite gain margin, with an exceptionally low unit-gain crossover frequency of 0.649 rad/s, ensuring overall system stability.

## 4. Experimental Results and Analysis

**Figure 11:**
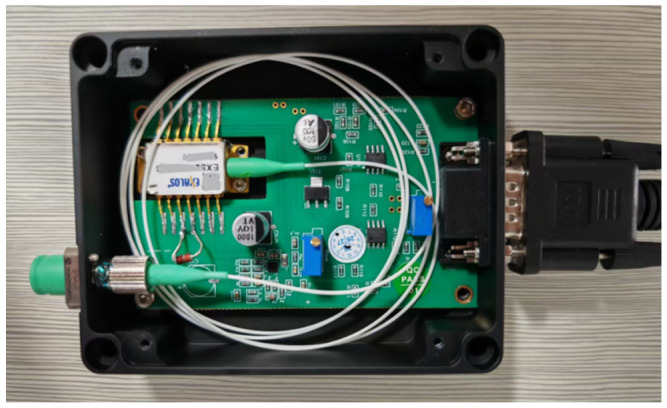
An internal view of the fabricated SLED light source module.

A SLED component with a central wavelength of 840 nm was selected, and the designed compact constant-current driving and temperature control circuits were implemented based on the aforementioned principles. An internal view of the fabricated SLED light source module is shown in Figure 11.

### 4.1. Test of Constant-Current Drive and Temperature Control System

**Figure 12:**
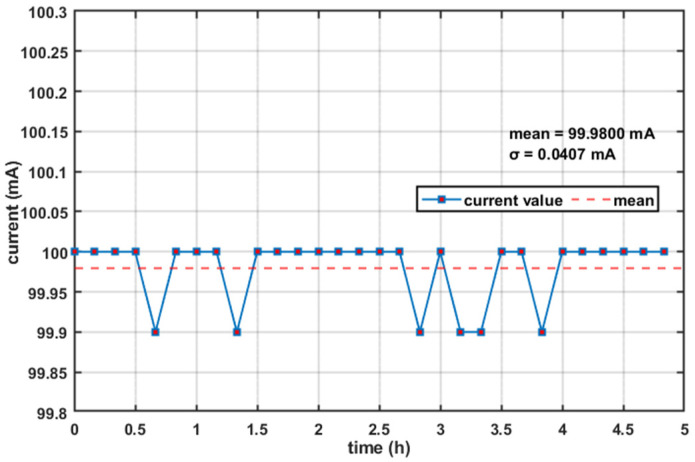
Drive current fluctuations over time.

Firstly, stability of the drive current was examined. In this experiment, the drive current was tuned to 100.0 mA under standard laboratory conditions at approximately 25 °C. The drive current was measured every 10 min with a multimeter and the source was kept emitting continuously for 5 h. The results are presented in Figure 12. Since the experimental data is measured by a multimeter, the accuracy is limited. The calculated average current was 99.98 mA, with a standard deviation of 0.0407 mA. The difference between the maximum and minimum drive currents was 0.1 mA. The fluctuation rate of the drive current was calculated using the formula:

(17) $$\gamma \;=\;\frac{\left(I_{max}\;-\;I_{min}\right)}{I_{set}}\times 100\%$$

where *I_set_* is the set value of the drive current. The calculated *γ* was 0.1%, corresponding to a fluctuation rate 0.1%.

**Figure 13:**
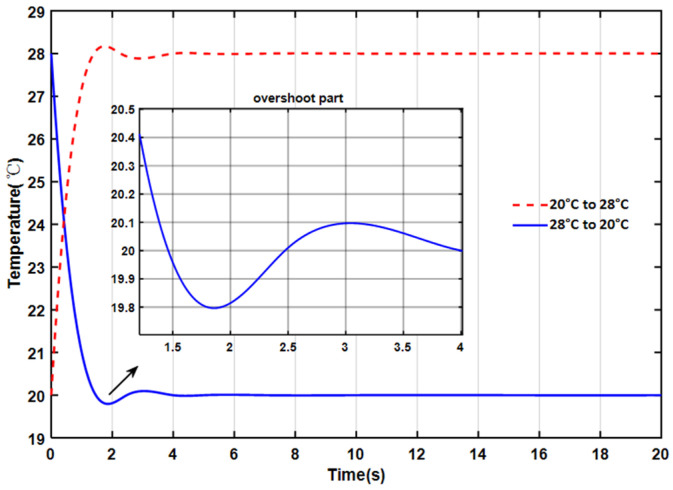
Temperature control stability.

To evaluate the temperature regulation performance of the thermal control system, the light source drive current was set to 100 mA, and the initial operating temperature was set to 28 °C. After the operating temperature stabilized, the setpoint was changed to 20 °C to test the system’s stability. A second test involved changing the setpoint from 20 °C to 28 °C. The temperature variation is shown in Figure 13. After changing the set temperature, the internal temperature of the light source rapidly approached the target value, reaching an extreme value in approximately 1.9 s, and then gradually stabilized at the set temperature. The maximum overshoot was about 0.2 °C, and the settling time was about 4.3 s. After the operating temperature stabilized, the actual temperature exhibited minor fluctuations around the setpoint, approximately 0.01 °C.

Furthermore, the ability of the thermal control system to maintain a stable internal operating temperature under different ambient temperatures was tested. A constant temperature chamber was used, with ambient temperatures sequentially set to 5 °C, 15 °C, 25 °C, 35 °C, and 45 °C, while humidity was held constant at 50%. After the chamber reached each set temperature, the module’s internal temperature were measured. As shown in Figure 8, the internal temperature exhibits a linear relationship with the OUT1 value, allowing direct conversion of OUT1 readings to internal temperatures via the calibration curve (The resistance value of the feedback resistor in the actual circuit is 50 kΩ). The light source, with its drive current set to 100 mA and operating temperature set to 22.5 °C, was placed inside the chamber. The test results are presented in Table 2. The results show that when the ambient temperature varied significantly (from 5 °C to 45 °C), the fluctuation range of the OUT1 value was 0.001 V, corresponding to a temperature fluctuation range of approximately 0.01 °C. According to the data sheet of the SLED component, fluctuations of 0.01 °C in operating temperature cause almost no change in the SLED chip’s power-current characteristics or spectral properties. Therefore, fluctuations of this magnitude do not affect the module’s suitability for OCT applications.

### 4.2. Test of Light Source Output

**Figure 14:**
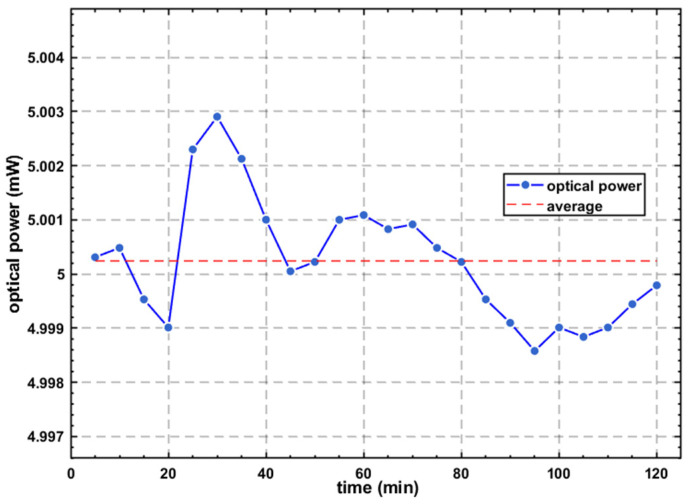
Optical power fluctuations over time.

The above experiments confirmed the proper functioning of the constant-current drive system and the temperature control system. Subsequently, the light source output was monitored. After the light source was turned on, a soft-start circuit allowed the drive current to increase gradually, and the output power reached near the set value within 5 s. Under indoor conditions, the light source output power was set to 5 mW and operated continuously for two hours. A optical power meter (PM60A, Thorlabs, Shanghai, China) was used to record the optical power value every 5 min. The test results are shown in Figure 14. The results show that the optical power variation was less than 0.005 mW, meeting the requirements for OCT applications.

**Figure 15:**
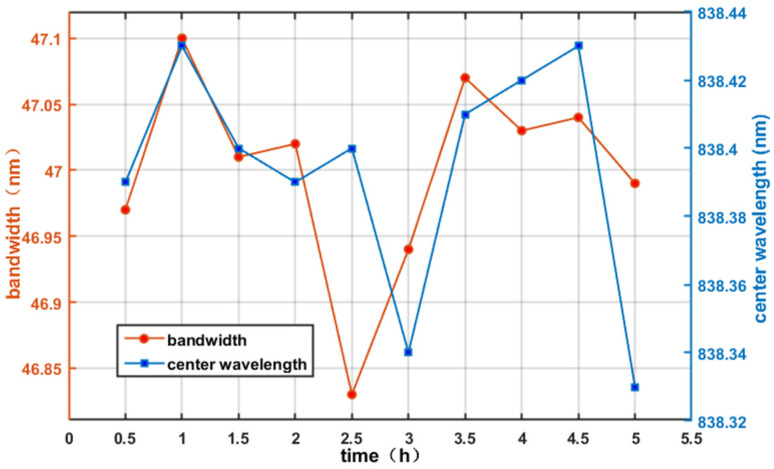
The changes in bandwidth and center wavelength over time.

**Figure 16:**
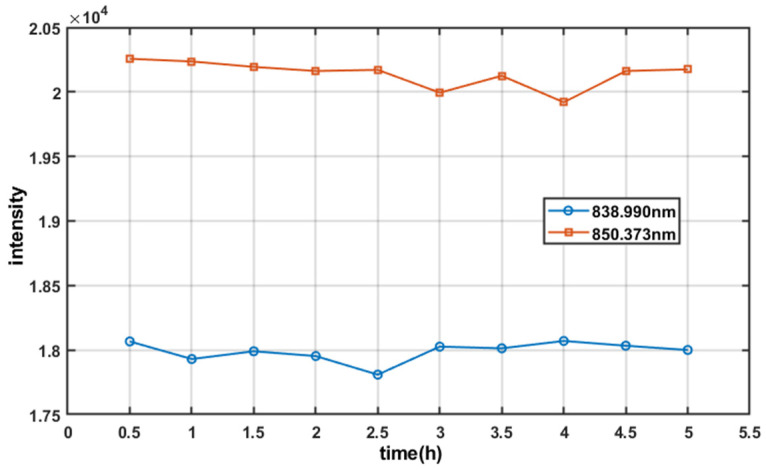
The changes in optical intensity at two wavelength points.

Next, the optical spectrum analyzer was used to monitor the spectral characteristics including bandwidth and ripple which may critically impact OCT performance. The spectral properties were recorded at 30 min intervals during 5 h operation. The fluctuations of spectral bandwidth (full width at half maxima) and central wavelength versus time are shown in Figure 15, the fluctuations of intensity values at 838.990 nm and 850.373 nm are shown in Figure 16. The results demonstrate that the spectral bandwidth variation range < 0.27 nm (fluctuation < 0.6%). The central wavelength was also stable, varying by ≤0.1 nm only.

The axial resolution, a decisive factor affecting the imaging clarity of an OCT system, can generally be expressed as:

(18) $$\delta_{z}\;=\;\frac{2\ln2}{\pi }\frac{\lambda_{0}^{2}}{\Delta \lambda }$$

where *λ*_0_ is the central wavelength and ∆*λ* is the spectral bandwidth. Based on the measured fluctuations in central wavelength and spectral bandwidth, the average axial resolution of the system was calculated to be 6.5992 µm, with a standard deviation of 0.0107 µm. This stability significantly surpasses the industry standard of 1%.

For each wavelength point (838.990 nm and 850.373 nm), the maximum and minimum optical intensities from ten measurements were substituted into the ripple calculation formula

(19) $$Ripple=10lg\left(\frac{P_{max}}{P_{min}}\right)$$

The ripple values at the two wavelength points are 0.063 dB and 0.073 dB, respectively. These results comply with the OCT application requirement of <0.2 dB while maintaining a substantial margin.

**Figure 17:**
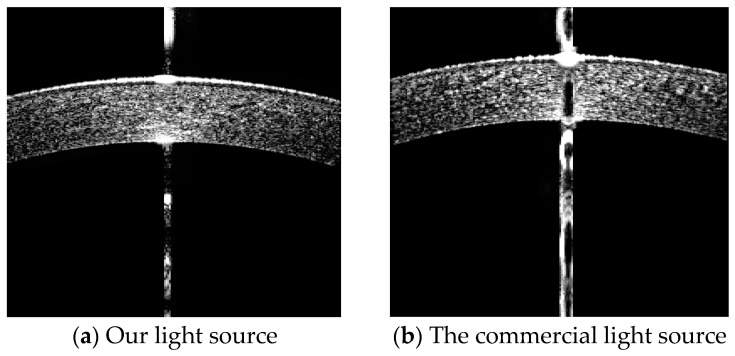
The corneal imagings of OCT.

The developed light source module was integrated into a commercial OCT ophthalmologic device (AXL-100, Ningbo Ming Sing Optical R&D Co., Ltd., Ningbo, China) for human corneal imaging. The same human eye was sequentially imaged using the device both before and after its light source was replaced. A comparison of the imaging results between the proposed light source and the original one is shown in Figure 17. The results indicate that the proposed light source provides imaging clarity comparable to that of the commercial source, enabling clear differentiation of ocular tissue layers. Moreover, image acquired with the proposed light source exhibits more regular contours and higher contrast.

## 5. Conclusions

In this study, a compact SLED constant-current drive and temperature control circuit were designed and implemented on the basis of hardware circuit design, control system mathematical model analysis and circuit simulation and verification. The constant-current drive circuit, based on the negative feedback principle, improves upon conventional current-limiting protection designs to achieve higher circuit integration. A miniature dedicated controller chip was utilized in the temperature control system, ensuring control accuracy while reducing the system volume. Through systematic modeling and Bode plot analysis of the compensation circuit, an effective PID compensation circuit was designed to precisely regulate the operating temperature. The module size is less than 10 × 8 × 3 cm^3^, including the casing, and it operates without computer support. Experimental results show that the system designed in this study achieves a driving current control accuracy of 0.1%. When the ambient temperature varies from 5 °C to 45 °C, the temperature control error is maintained within 0.01 °C. At the same time, the measured fluctuation range of the output optical power is less than 0.005 mW. According to the measured fluctuations in central wavelength and spectral bandwidth, the average axial resolution of OCT was calculated to be 6.5992 µm, with a standard deviation of 0.0107 µm. This stability is significantly better than the industry standard of 1%. The comparison between our scheme and other schemes is shown in Table 3. Compared with the currently advanced schemes, our scheme achieves similar control accuracy, while offering significant advantages in terms of size, cost, power consumption, and complexity.

Overall, the constant-current drive and temperature control system designed in this study greatly reduces the complexity and cost of the system while ensuring control accuracy, and thus has practical significance.

## Figures and Tables

**Table 1:** Parameter values of compensation circuit.

*R_P_*	*R_I_*	*R_D_*	*C_I_*	*C_D_*	*C_F_*
43 kΩ	160 kΩ	160 kΩ	10 μF	0.01 μF	0.01 μF

**Table 2:** Operating temperatures under different ambient temperatures.

Ambient Temperature (°C)	5	15	25	35	45
OUT1 (V)	1.067	1.067	1.067	1.067	1.068
Operating Temperature (°C)	22.50	22.50	22.50	22.50	22.51

**Table 3:** Evaluation of our scheme vs. other schemes.

Characteristics	Schemes		
	Our Scheme	Microcontroller Scheme [13,15,16]	FPGA Scheme [18]
Composition	Fully integrated single-board design	Dual-component Two driver boards + MCU	Dual-component Two driver boards + FPGA
BOM Cost	Most cost-effective (about 200 CNY)	Moderately higher cost (about 300 CNY)	Highest cost (about 600 CNY)
Power Consumption	Most energy-efficient (about 1 W)	Moderate power consumption (about 2.5 W)	Higher power consumption (about 5 W)
Control Accuracy	High (Temperature fluctuation: 0.01 °C)	High (Temperature fluctuation: 0.01 °C)	High (Temperature fluctuation: 0.01 °C)
Implementation Complexity	Simplest (Hardware-based closed-loop control)	Requires software development	Requires complex HDL development

## Data Availability

The original contributions presented in this study are included in the article. Further inquiries can be directed to the corresponding authors.

## Abbreviations

The following abbreviations are used in this manuscript:

SLED	Superluminescent light-emitting diode
OCT	Optical coherence tomography
FPGA	Field-programmable gate array
NTC	Negative temperature coefficient
PID	Proportional-integral-derivative
